# Identification and Functional Analysis of ncRNAs Regulating Intrinsic Polymyxin Resistance in Foodborne *Proteus vulgaris*

**DOI:** 10.3390/microorganisms12081661

**Published:** 2024-08-13

**Authors:** Hongyang Zhang, Tao Wu, Haihua Ruan

**Affiliations:** Tianjin Key Laboratory of Food Biotechnology, School of Biotechnology and Food Science, Tianjin University of Commerce, Tianjin 300134, China; zhyang@tjcu.edu.cn (H.Z.); wutao@tjcu.edu.cn (T.W.)

**Keywords:** *Proteus vulgaris*, non-coding RNA, target mRNA, polymyxin resistance, screening

## Abstract

Polymyxin, known as the “last line of defense” against bacterial infection, exerts a significant inhibitory effect on a wide range of Gram-negative pathogenic bacteria. The presence of strains, specifically *Proteus vulgaris* species, displaying intrinsic polymyxin resistance poses significant challenges to current clinical treatment. However, the underlying mechanism responsible for this intrinsic resistance remains unclear. Bacterial non-coding RNAs (ncRNAs) are abundant in genomes and have been demonstrated to have significant regulatory roles in antibiotic resistance across various bacterial species. However, it remains to be determined whether ncRNAs in *Proteus vulgaris* can regulate intrinsic polymyxin resistance. This study focused on investigating the foodborne *Proteus vulgaris* strain P3M and its intrinsic polymyxin resistance regulation mediated by ncRNAs. Through a combination of bioinformatics analysis, mutant construction, and phenotypic experimental verification, we successfully identified the ncRNAs involved and their potential target genes. These findings serve as an essential foundation for the precise identification of ncRNAs participating in the intricate regulation process of polymyxin resistance. Additionally, this study offers valuable insights into the efficient screening of bacterial ncRNAs that contribute positively to antibiotic resistance regulation.

## 1. Introduction

The widespread and frequent use of various types of antibiotics creates repetitive selective pressures in the environment. This continuous exposure leads to the emergence of multidrug-resistant bacteria, significantly reducing the available choices for effective clinical treatments [1,2,3]. According to reports from the World Health Organization, approximately 700,000 individuals worldwide lose their lives each year due to diseases caused by multidrug-resistant bacteria. Polymyxin, an essential type of polypeptide antibiotic, has traditionally been regarded as the “last line of defense” against these resistant strains [4,5,6]. Since the early 1940s, scientists have identified five distinct polymyxins (A-E). Among them, polymyxin B and colistin E have gained widespread usage in clinical settings for treating infections caused by multidrug-resistant Gram-negative bacteria [7,8,9,10].

The extensive use of polymyxin in clinical treatment, agricultural production, and animal husbandry has resulted in a decline in its effectiveness against Gram-negative bacteria. Alarmingly, the emergence of drug-resistant strains has been observed [11,12]. The complete mechanism of bacterial polymyxin resistance is complex and exhibits intricate characteristics [13,14]. Furthermore, certain bacteria, such as *Providencia*, *Serratia*, and *Proteus*, exhibit intrinsic resistance to polymyxin, meaning that this resistance is inherent and not acquired through mutation or the horizontal transfer of resistance genes [13,15,16]. Complicating matters further, many of these resistant strains are clinical pathogens [17,18].

*Proteus vulgaris*, a well-known polymyxin-resistant strain, belongs to the Enterobacteriaceae family and *Proteus* genus [19]. It is a prevalent foodborne bacterium found in various environmental sources such as spoiled food and the intestinal tracts of aquatic animals [20,21]. *Proteus vulgaris* easily accumulates and transfers through the food chain, causing gastrointestinal and urinary tract infections [22]. Currently, the mechanism behind the intrinsic polymyxin resistance in *Proteus vulgaris* species is not fully understood. Baron et al., in 2018, suggested that the constitutive activation of the *arn* operon and the addition of 4-amino-4-deoxyl-arabinose (L-Ara4N) contribute to the intrinsic resistance to polymyxin in *Proteus vulgaris* [23]. Subsequently, Bakthavatchalam et al. summarized the commonly known mechanisms of intrinsic polymyxin resistance in a review study. They found that the constitutive expression of the *arnBCADTEF* operon and the *eptB* gene led to the addition of phosphoethanolamine (pEtN) and L-Ara4N cationic groups to the bacterial lipopolysaccharide, enhancing the stability of the outer membrane [24]. This modification increases the net charge of the lipopolysaccharide and reduces the binding of polymyxins, resulting in intrinsic resistance among strains [18,25,26].

The regulatory mechanism of bacterial drug resistance, formed through long-term evolution, is complex, similar to other physiological regulatory processes in bacteria. Numerous studies have demonstrated the involvement of various regulatory factors in the regulation of bacterial resistance. For instance, TetR, a common transcription regulatory factor in Gram-negative bacteria, regulates tetracycline resistance by binding to the promoter region of the tetracycline resistance gene *tet(A)* [27]. In *Pseudomonas aeruginosa*, the regulatory factor CpxR directly activates the RND efflux pump MexAB-OprM, contributing to multidrug resistance [28]. Based on these findings, it is plausible to speculate that similar regulatory factors may also exist in inherently polymyxin-resistant strains like *Proteus vulgaris*. Non-coding RNA (ncRNA) is one such important regulatory factor with potential involvement.

Currently, various types of ncRNAs with significant regulatory functions in drug resistance have been identified in bacteria such as *Escherichia*, *Pseudomonas*, and *Salmonella* [29,30,31]. In the absence of antibiotics, these ncRNAs can inhibit the expression of drug-resistant genes by competitively binding to the ribosome-binding site (RBS) or by creating transcriptional terminators in the 5′ untranslated region (UTR) of the target gene. However, when antibiotics are present, the secondary structure of ncRNAs changes, leading to different base-pairing patterns that do not impede transcription or translation processes. Consequently, the expression of drug-resistant genes is activated [32,33]. Despite these findings, there is a limited amount of research on the regulatory role of bacterial ncRNAs in polymyxin resistance.

In this study, we focused on the previously identified multidrug-resistant *Proteus vulgaris* strain P3M as our research subject. Our aim was to explore and preliminarily identify ncRNAs that directly and positively regulate the intrinsic polymyxin resistance of this strain, along with their potential target genes. Additionally, we analyzed the evolutionary conservation characteristics of these ncRNAs among *Proteus vulgaris* species. This study aims to facilitate effective research on bacterial drug resistance, complementing specific experimental approaches.

## 2. Materials and Methods

### 2.1. Strains and Cultural Conditions

The strains and plasmids utilized in this study are listed in Table 1. All strains were cultured in Luria–Bertani (LB) medium (DINGGUO, Beijing, China) at a temperature of 37 °C. The medium was supplemented with tetracycline (10 µg/mL) (DINGGUO, Beijing, China), streptomycin (50 µg/mL) (DINGGUO, Beijing, China), and polymyxin B (1024 mg/L) (DINGGUO, Beijing, China) as required.

### 2.2. Construction of ncRNA34 Mutant Strain

To assess the function of ncRNA34 in regulating polymyxin resistance, deletion mutant strains were generated through homologous recombination. The genomic DNA of *P. vulgaris* strain P3M was extracted and used as a template to amplify the upstream and downstream homologous fragments. These fragments were then fused using PCR to obtain a fusion fragment, which was subsequently verified and ligated into the suicide plasmid pEX18Tc [35]. The resulting recombinant plasmids were transformed into *E. coli* DH5α competent cells, and correct plasmids were extracted and transferred into *E. coli* S17 [34]. The correct *E. coli* S17 transformants were then introduced into the wild-type P3M strain through bi-parental mating. The mutant strains were screened using homologous recombination single and double crossovers and were finally confirmed through colony PCR and sequencing.

### 2.3. Construction of ncRNA34 Complemented Strain

The ncRNA34 deletion mutant strain was used as a basis to construct the ncRNA34 complemented strains. The complete sequence of ncRNA34, including both the promoter and terminator regions, was amplified using the P3M genome as a template and subsequently ligated into the cloning vector pDN18 [36]. The resulting recombinant plasmids were then transferred into *E. coli* DH5α competent cells for verification through colony PCR and sequencing. Once correct plasmids were confirmed, they were transferred into *E. coli* S17. The correct *E. coli* S17 transformants were then subjected to bi-parental mating with the ncRNA34 deletion mutant strain. Positive transformants were subsequently screened and verified through colony PCR and sequencing to ensure the successful construction of the ncRNA34 complemented strains.

### 2.4. Determination of the Minimum Inhibitory Concentration (MIC)

The minimum inhibitory concentrations (MICs) of the wild-type strain P3M, deletion mutant strain ∆ncRNA34, and its complemented strain com-ncRNA34 were determined using the broth microdilution method, following the recommendations of the Clinical and Laboratory Standards Institute (CLSI) [37].

### 2.5. Spot Growth Assays

The survival rate of P3M and its derivatives in response to polymyxin B was determined following the described procedure [38,39]. The strains were initially cultured overnight in LB broth at 37 °C and subsequently transferred into fresh LB broth, allowing them to reach an *OD*_600_ of 0.6. The bacterial solution obtained was then transferred into fresh LB broth, with or without the addition of 1024 mg/L polymyxin B, and incubated for 30 min. Gradient dilution was performed on the treated cultures, and 5 µL droplets were spotted onto LB agar plates. These plates were then incubated at 37 °C for 24 h until colony growth was observed.

### 2.6. Quantitative Real-Time PCR (qRT-PCR)

Total RNA from P3M and ∆ncRNA34 was isolated using a Bacteria RNA Extraction Kit (Vazyme, Nanjing, China) and subsequently reverse-transcribed into cDNA. The qRT-PCR reaction was performed using a reaction system consisting of 1 µL of template cDNA (100 ng/µL), 10 µL of 2 × SYBR Green qPCR Master Mix (Vazyme, Nanjing, China), 1 µL of upstream primer and 1 µL of downstream primer (10 µM), and 7 µL of ddH_2_O, following the manufacturer’s instructions. Gene-specific primers, as listed in Table S1, were designed based on the genome sequence of P3M, and the 16S rRNA gene was used as an endogenous reference gene for normalizing the expression of target genes in each cDNA template.

### 2.7. RNA Secondary Structure Prediction

The secondary structures of candidate ncRNAs and potential target mRNAs in this study were predicted using the Mfold web server, following the provided instructions [40].

### 2.8. ncRNA Target Prediction

The interaction between CsiR and its potential target mRNA was predicted using the RNAup server, as described in references [41,42].

## 3. Results

### 3.1. The Potential Role of ncRNAs in the Regulation of Polymyxin Resistance in Proteus vulgaris

Research has shown that *Proteus vulgaris* exhibits intrinsic resistance to polymyxin, belonging to the group of polypeptide antibiotics [16]. In this study, we focused on investigating the polymyxin resistance of the *Proteus vulgaris* strain P3M, a foodborne multidrug-resistant bacterium isolated from *Penaeus vannamei*. Specifically, we examined its resistance to colistin E (CT) and polymyxin B (PB), which are typical representatives of polymyxins (Table 2). According to the CLSI drug breakpoint criterion, *Proteus vulgaris* is considered polymyxin-resistant when the minimum inhibitory concentration (MIC) of these two antibiotics reaches or exceeds 16 mg/L [37]. In this study, we discovered that even when the concentration of the two drugs reached the upper limit set by our experiment (1024 mg/L), the growth of the P3M strain remained unaffected. This finding suggests that P3M exhibits robust intrinsic polymyxin resistance.

**Figure 1:**
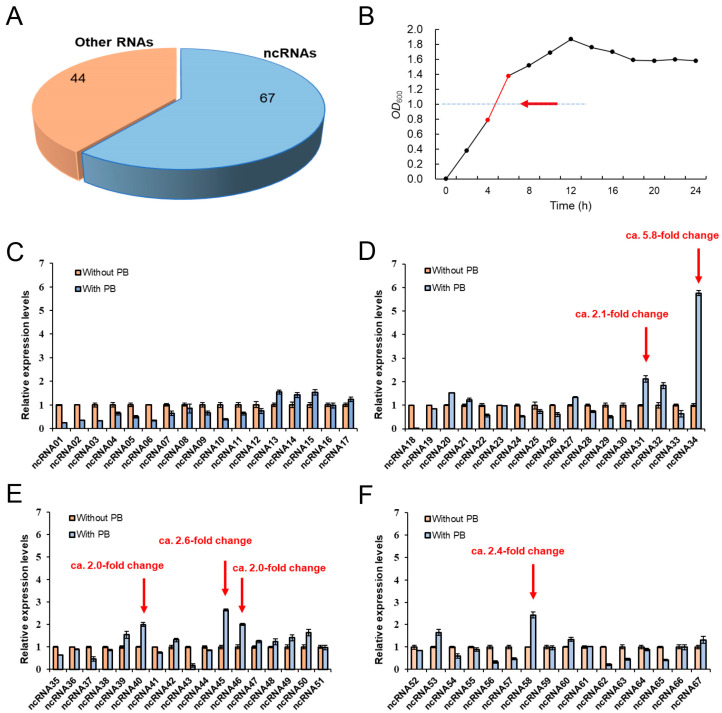
Screening and expression of ncRNAs in P3M under polymyxin stress. (**A**) Distribution of ncRNAs in P3M based on their numbers. (**B**) Growth curve of P3M, with the red line and arrow indicating the culture stage used for RNA extraction. (**C**–**F**) Relative expression levels of ncRNAs under polymyxin B treatment condition (1024 mg/L) compared to the control group.

In previous studies [19], a total of 111 RNAs were identified in the genome of P3M, out of which 67 belong to the category of ncRNAs (Figure 1A). In this study, we examined the expression of all 67 ncRNAs in LB liquid culture supplemented with polymyxin B (1024 mg/L). RNA extraction was conducted from bacteria in the logarithmic growth phase (*OD*_600_ = 1.0) (Figure 1B), and the expression levels of ncRNAs were assessed. Comparing the results with the control group without polymyxin treatment, we observed varying changes in the expression of the 67 ncRNAs. Our objective was to determine if there are ncRNAs that directly employ positive regulatory strategies in the regulation of polymyxin resistance in the *Proteus vulgaris* strain P3M. Consequently, we screened six ncRNAs (ncRNA31, ncRNA34, ncRNA40, ncRNA45, ncRNA46, and ncRNA58) that exhibited a significant up-regulated trend (fold change > 2) in the experimental group. The results suggested that these ncRNAs may play crucial positive regulatory roles in the modulation of intrinsic polymyxin resistance in P3M (Figure 1C–F).

### 3.2. Prediction of ncRNA Secondary Structure and Potential Target Genes

To investigate the regulatory function of the six candidate ncRNAs obtained earlier in polymyxin resistance, a functional identification process was required. Typically, this involves constructing deletion mutant strains and conducting phenotypic validation experiments. However, considering the time- and cost-intensive nature of knocking out all six candidate ncRNAs and validating their phenotype changes, it was more effective to narrow down the target range using bioinformatics prediction and analysis. ncRNAs exhibit complex secondary structures, often characterized by stem–loop regions that are essential for their regulatory functions [43,44,45]. To gain insights into the correlation between the structures of the six ncRNAs and their potential regulatory functions, we obtained their most likely folding forms based on minimum free energy (Figures S1–S6) [40]. These six ncRNAs display distinct secondary structures, but further research is needed to determine whether all of them play key roles in the regulation of polymyxin resistance and their associated target genes.

Numerous studies have demonstrated that ncRNAs typically exert regulatory functions through binding with target gene mRNA [38,39,46]. Hence, screening for target genes that can interact with the aforementioned six candidate ncRNAs is crucial to narrow down the research scope. Previous sequencing analyses revealed the presence of 218 antibiotic resistance genes (ARGs) in P3M [19]. Based on sequence alignment and gene function cluster analysis, 13 ARGs were determined to most likely be associated with polymyxin resistance (Table 3). The functional annotations of these genes were statistically analyzed using whole-genome sequencing and BLAST analysis. Most of these genes encode functional proteins such as hydrolases, dehydrogenases, and phosphotransferases, which are known to play significant roles in the efficient excretion and degradation of antibiotics within cells (Table 3). Thus, if the candidate ncRNAs have target genes directly involved in the regulation of polymyxin resistance, they are more likely to be found among these 13 ARGs.

**Figure 2:**
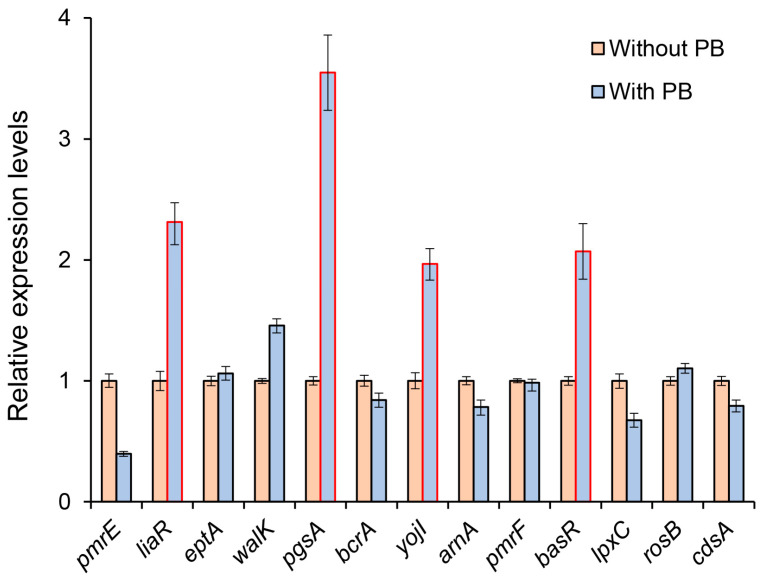
Relative expression levels of polymyxin resistance genes with or without polymyxin B treatment condition (1024 mg/L).

To investigate the potential involvement of the aforementioned ARGs in the regulation of polymyxin resistance mediated by ncRNAs, we examined the expression levels of these genes in response to polymyxin B treatment. Figure 2 illustrates the expression patterns of the genes when exposed to 1024 mg/L polymyxin B. It was observed that most genes did not show significant changes in expression compared to the untreated group. However, the expressions of *liaR*, *pgsA*, *yojI*, and *basR* were significantly up-regulated, with fold changes greater than 2 (marked in red boxes in Figure 2). Notably, *pgsA* exhibited the most significant up-regulation, with approximately a 3.5-fold increase in expression. These findings suggest that these four genes are highly responsive to signals generated under the antibiotic stress induced by polymyxin B. Based on these observations, we can preliminarily infer that the target genes for the candidate ncRNAs are likely to be among these four genes, which exhibit evident expression changes.

**Figure 3:**
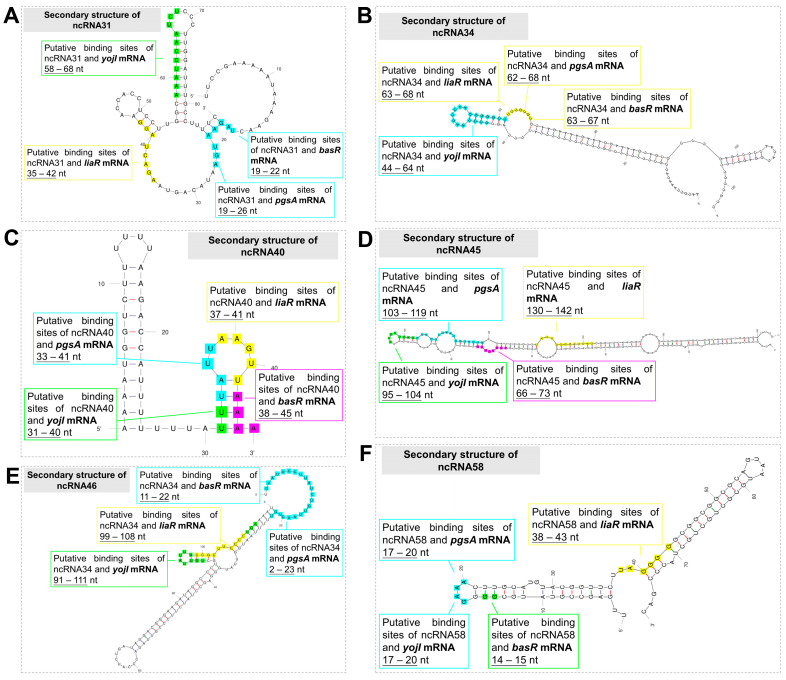
Secondary structures of ncRNAs and interaction sites with potential target genes.

Previous research has indicated that ncRNAs typically exert regulatory functions by interacting with specific target mRNAs, often through binding sites located within the stem–loop regions of ncRNAs [38,39]. In order to investigate the potential interactions between the six candidate ncRNAs and the four selected target genes identified in the preliminary screening, we performed binding-site prediction analysis. In Figure 3, the binding sites of the four target gene mRNAs on the ncRNAs are highlighted using different colors. Additionally, we have marked the corresponding interaction sites on the target gene mRNAs with distinct colors at their respective positions (Figures S7–S10). These visual representations provide insights into the potential binding and regulatory interactions between the candidate ncRNAs and their target gene mRNAs.

Previous research has identified two key factors that contribute to the regulatory roles of ncRNA-mRNA interactions: (1) the presence of interaction sites on the ncRNA, typically located within the stem–loop structure, and (2) the ability of the ncRNA-mRNA binding to facilitate the improved translation of the target gene mRNA at the post-transcriptional level. This is achieved by unfolding specific hairpin structures within the mRNA, thus reducing steric hindrance. In Table 4, we present the results of the interaction analysis between the six candidate ncRNAs and the four potential target gene mRNAs based on cross-screening using the aforementioned factors. The analysis reveals that ncRNA34 and ncRNA45 show a tendency to interact with *pgsA* mRNA and *yojI* mRNA, suggesting their potential regulatory roles in the translation process of these target genes.

**Figure 4:**
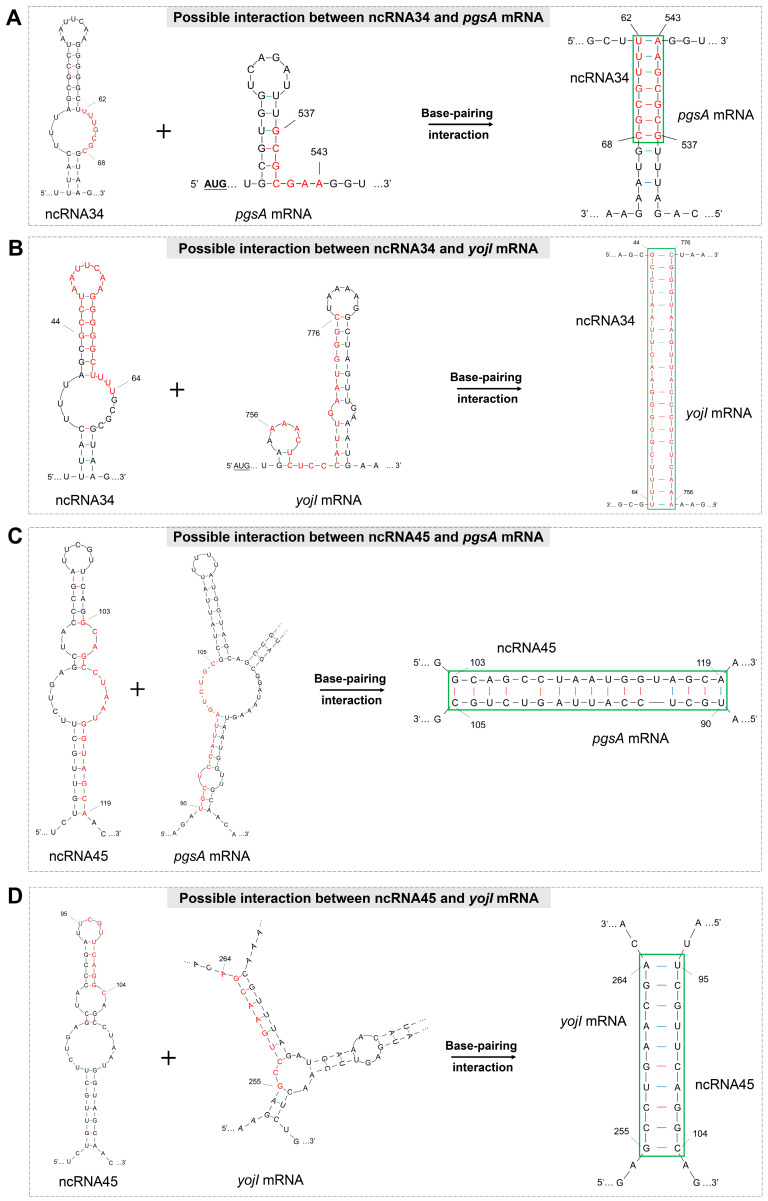
Schematic representation of the base-pairing complex formation between the base-pairing sites of ncRNA34/45 and the complementary sequence of *pgsA*/*yojI* mRNA.

To gain a better understanding of the resulting binary complexes and structural changes that occur during the interaction between ncRNA34, ncRNA45, and their respective target mRNAs, we depicted the binding sites of these ncRNAs with the target mRNAs in an interaction model diagram, as shown in Figure 4. The interaction model diagram demonstrates that the formation of complexes involving ncRNA34 and ncRNA45 effectively unfolds the stem–loop structures located at or near the action sites on the target mRNAs. This suggests that the interaction between these two ncRNAs and the target gene mRNAs has the potential to positively regulate the polymyxin resistance of P3M. The unfolding of the stem–loop structures may facilitate a better translation process of the target gene mRNAs, ultimately leading to enhanced resistance against polymyxin.

These visual representations provide valuable insights into the structural dynamics of ncRNA-mRNA interactions and their potential impact on polymyxin resistance. Further experimental studies are warranted to confirm these findings and elucidate the precise molecular mechanisms underlying the regulatory roles of ncRNA34 and ncRNA45 in the context of polymyxin resistance in P3M.

### 3.3. Function Determination of Candidate ncRNAs

**Figure 5:**
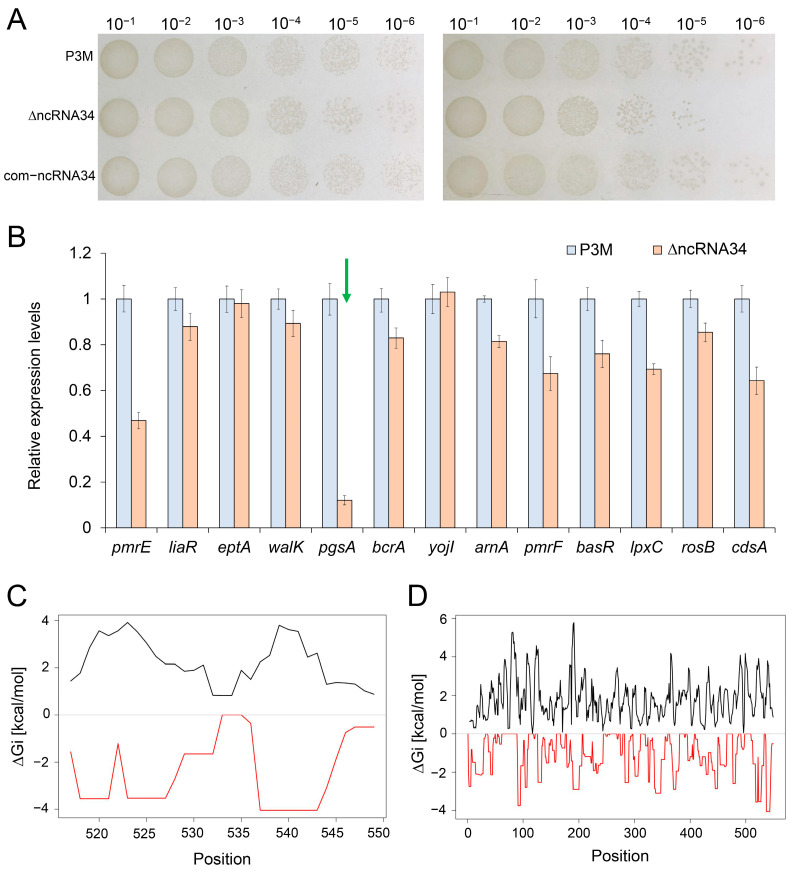
Functional identification of ncRNA34 and its possible target genes. (**A**) Spot growth assays of P3M, ΔncRNA34, and com-ncRNA34 under polymyxin B treatment condition. (**B**) Relative expression levels of polymyxin resistance genes in wild-type P3M and ∆ncRNA34. (**C**,**D**) The interaction free energy (RED) ∆Gi and the energy needed to open existing structures in *pgsA* mRNA sequence (BLACK) for the target region (**C**) and the whole range (**D**).

Based on the aforementioned analysis results, the roles of ncRNA34 and ncRNA45 in the regulation of polymyxin resistance were investigated by constructing mutant strains. However, contrary to our initial hypothesis, the absence of ncRNA45 did not result in a reduction in polymyxin resistance in the strains. Only the absence of ncRNA34 showed a certain regulatory effect. It is important to note that the intrinsic resistance background of P3M to polymyxin is already high, making it challenging to observe numerical changes in MIC values. This is why the MIC of polymyxin B was not significantly reduced in the absence of ncRNA34 (Table 5). Nonetheless, it is evident that ncRNA34 plays a specific role in positively regulating polymyxin resistance, as depicted in Figure 5A.

Upon the deletion of ncRNA34, the expression of nearly all polymyxin resistance-related genes showed varying degrees of down-regulation, although not at a statistically significant level. Among these genes, the most pronounced expression change was observed in the *pgsA* gene, with its expression level reduced to only about 10% of the initial level (marked in green arrow in Figure 5B). Conversely, the expression of the *yojI* gene in the mutant strains exhibited minimal difference compared to that in the wild type, suggesting that there is no direct regulatory relationship between ncRNA34 and *yojI* mRNA. These observations strongly suggest that *pgsA*, rather than *yojI*, is the most likely target gene directly regulated by ncRNA34. Furthermore, the analysis of the interaction free energy between ncRNA34 and *pgsA* mRNA (Figure 5C,D) indicates a favorable binding stability, supporting the potential regulatory interaction between ncRNA34 and *pgsA* mRNA.

These findings provide valuable insights into the specific regulatory role of ncRNA34 and its potential influence on the expression of polymyxin-resistance-related genes, particularly *pgsA*. Future research should aim to elucidate the intricate mechanisms involved in the ncRNA34-*pgsA* mRNA interaction to gain a comprehensive understanding of their functional relationship.

### 3.4. Evolutionary Conservation Analysis of ncRNA34-pgsA mRNA Interaction

**Figure 6:**
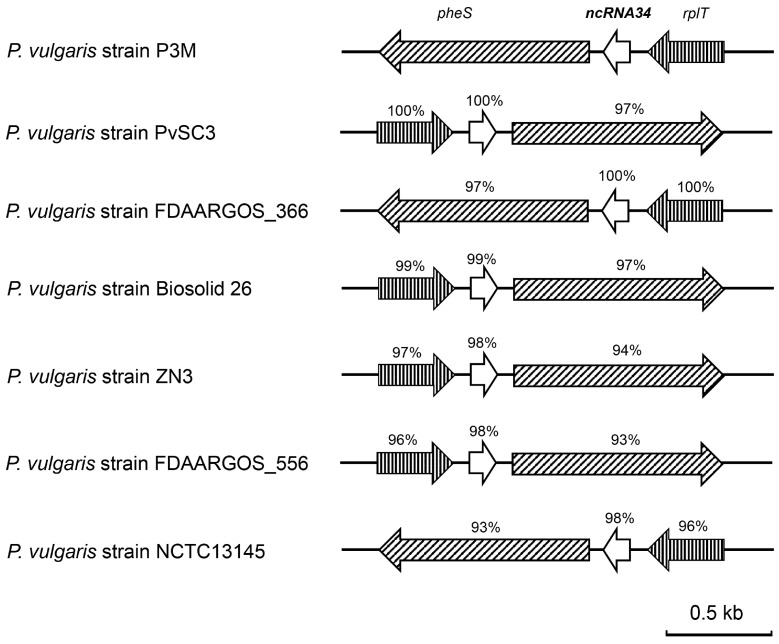
Genomic organization of the P3M ncRNA34 gene and comparison with equivalent loci from other sequenced *P. vulgaris* strains.

**Figure 7:**
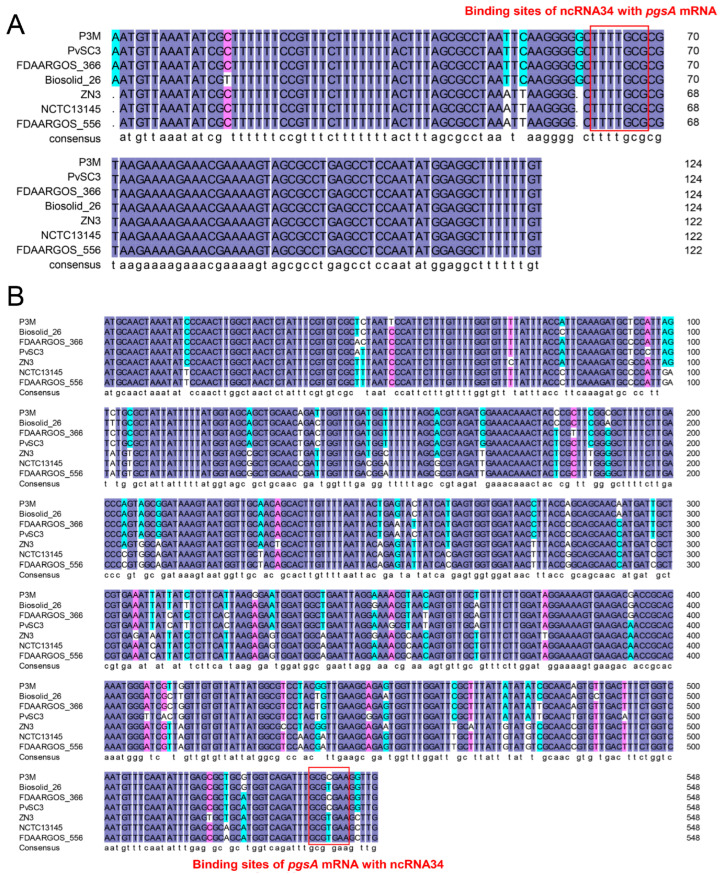
Sequence alignment of ncRNA34 (**A**) and *pgsA* (**B**) in P3M with homologous sequences in all sequenced *P. vulgaris* strains, respectively. Sequences marked with red boxes were the binding sites.

The sequence alignment results revealed that ncRNA34 was only present in the seven completely whole-genome-sequenced *Proteus vulgaris* strains. This indicates that ncRNA34 is a species-specific regulatory element. As depicted in Figure 6 and Figure 7A, ncRNA34 exhibited a high degree of sequence conservation across all seven strains, with a similarity of over 97%. Although the transcription direction of ncRNA34 was not completely consistent, the surrounding genes, *pheS* and *rplT*, also displayed high sequence conservation. Furthermore, the sequence of the putative target gene, *pgsA*, showed significant consistency among the analyzed strains (Figure 7B).

To verify the intraspecific conservation of potential interaction mechanisms, a sequence analysis was conducted to examine the consistency of the ncRNA34 and *pgsA* mRNA binding sites in Proteus vulgaris strains. As depicted by the red boxes in Figure 7A,B, the binding sites on ncRNA34 and *pgsA* mRNA exhibited high conservation across the seven *Proteus vulgaris* strains. Specifically, the sequences of the binding sites on ncRNA34 showed 100% consistency among all strains. In the case of the binding sites on *pgsA* mRNA, there was a minor variation observed in the Biosolid_26, ZN3, NCTC13145, and FDAARGOS_556 strains, where cytosine (C) was replaced by thymine (T). However, this single base change did not appear to affect the predicted interactions. In the actual interaction process, guanine–cytosine (G-C) pairing can indeed be replaced by guanine–uracil (G-U) pairing.

Further studies are required to investigate the specific mechanisms and functional consequences of ncRNA34 and *pgsA* mRNA interaction, considering the observed sequence conservation within different strains of *Proteus vulgaris*.

## 4. Discussion

The regulation of antibiotic resistance in bacteria mediated by ncRNAs offers several advantages, including rapid response, flexible and precise regulation, and minimal metabolic burden. Understanding the regulatory pathways involved in drug resistance provides an opportunity for the sequence-specific inhibition of these ncRNAs through programmable RNA-targeted therapy in clinical settings. This approach holds potential to effectively treat infections caused by drug-resistant bacteria [47,48].

Considering the critical role of polymyxin as the “last line of defense” against drug-resistant bacteria, elucidating the molecular mechanisms of ncRNA in the regulation of intrinsic polymyxin resistance in common pathogenic bacteria is crucial. Such knowledge will serve as a vital foundation for the development of new RNA-targeted antibacterial drugs and the effective application of RNA-targeted therapies. By doing so, we can significantly delay the onset of the “post-antibiotic era” and combat the challenges posed by antibiotic resistance in a more effective manner.

In this study, we conducted a preliminary screening to identify ncRNA34 among six candidate ncRNAs that exhibited a positive response to polymyxin-induced conditions. Additionally, we identified a potential target gene, *pgsA*, which encodes CDP-diacylglycerol-glycerol-3-phosphate-3-phosphatidyltransferase and is associated with polymyxin resistance in the *Proteus vulgaris* strain P3M. Furthermore, through binding-site analysis, we determined that the interaction pattern between ncRNA34 and *pgsA* mRNA is highly conserved within the *Proteus vulgaris* species. This finding suggests the evolutionary significance of the ncRNA-mediated regulatory mechanism in polymyxin resistance within *Proteus vulgaris* species.

Indeed, to gain a comprehensive understanding of the interaction mechanism between ncRNA34 and *pgsA* mRNA, further investigations are necessary in subsequent studies. This may involve constructing *pgsA* deletion mutant strains and complemented strains, validating changes in MIC, analyzing the binding characteristics of ncRNA34 and *pgsA* mRNA, and assessing post-transcriptional stability. These additional experimental tasks are essential to further validate the conclusions drawn from this study.

**Figure 8:**
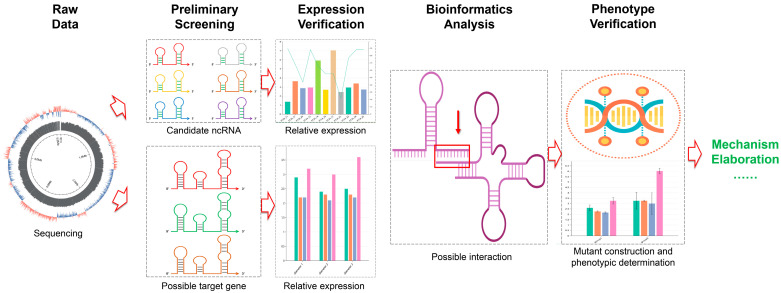
Schematic diagram of screening and preliminary identification of ncRNAs and target genes regulating polymyxin resistance in this study.

The findings of this study not only reveal, for the first time, the role of ncRNA34 in regulating polymyxin resistance in *Proteus vulgaris* from the perspective of ncRNA, but also establish a screening method that effectively narrows the research scope through bioinformatics analysis, providing significant theoretical guidance (Figure 8). The method employed in this study is particularly suitable for directly screening ncRNAs that have positive regulatory roles, as demonstrated by the successful identification of ncRNA34 and its potential target gene, *pgsA*. However, it is important to note that the other five candidate ncRNAs, although not identified as the direct regulators, cannot be completely ruled out due to their significant expression changes under polymyxin B treatment conditions. It is possible that these ncRNAs may have indirect regulatory roles in the process of polymyxin resistance regulation, and the corresponding target genes may have a broader range for screening. Further investigations are required to explore these possibilities and expand our understanding of the regulatory mechanisms involved.

Furthermore, it is important to note that, in this study, our focus was solely on identifying ncRNAs with potential positive regulatory roles. However, considering the expression results of the ncRNAs shown in Figure 1C–F, it is highly likely that there are also ncRNAs that exhibit negative regulatory functions. An in-depth identification and characterization of these ncRNAs is necessary to establish and enhance our understanding of the global network of ncRNAs involved in regulating intrinsic polymyxin resistance in *Proteus vulgaris*. This comprehensive approach will contribute to a more complete understanding of the regulatory mechanisms at play in polymyxin resistance.

## 5. Conclusions

The foodborne *Proteus vulgaris* strain P3M exhibits polymyxin resistance, posing a significant threat to food safety and human health. This study utilizes bioinformatics analysis and phenotypic identification experiments to reveal the pivotal role of ncRNA34 in regulating polymyxin resistance in P3M. The potential target gene associated with this regulatory function is identified as *pgsA*. This study investigates the ncRNA perspective to explore potential pathways of polymyxin resistance regulation in foodborne Proteus vulgaris, providing critical insights that can inform policy decisions in the interest of food safety and public health. Moreover, a time-efficient ncRNA screening strategy is established, facilitating the identification of ncRNAs with positive regulatory functions and their target genes. Future research will focus on exploring the interaction mechanism between ncRNA34 and its putative target gene *pgsA*, aiming to further comprehend the regulatory mechanisms underlying polymyxin resistance in *Proteus vulgaris* species.

## Figures and Tables

**Table 1:** Strains and plasmids used in this study.

Strains or Plasmids	Characteristics	Sources
*P. vulgaris* strains		
P3M	Wild-type strain	[19]
∆ncRNA34	ncRNA34 deletion mutant strain	This study
com-ncRNA34	ncRNA34 complemented strain, Tc^r^	This study
*E. coli* strains		
*E. coli* DH5α	Competent cell for cloning	CWBIO Company
*E. coli* S17	Mobilizing donor strain, Sm^r^	[34]
Plasmids		
pEX18Tc	Suicide plasmid used for constructing the deletion mutant strain, Tc^r^	[35]
pDN18	Broad-spectrum clone plasmid used for the construction of functional complemented strain, Tc^r^	[36]

**Table 2:** Polymyxin resistance of *Proteus vulgaris* strain P3M.

Antibiotic	MIC (mg/L), Interpretation
Colistin E (CT)	>1024, Resistant
Polymyxin B (PB)	>1024, Resistant

**Table 3:** Possible polymyxin resistance genes in P3M.

No.	Gene Name	Function	Location ^a^	Strand ^b^
1	*pmrE*	Nucleotide sugar dehydrogenase	54448–55614	−
2	*liaR*	Response regulator	421750–422412	−
3	*eptA*	Metal-dependent hydrolase	435097–436788	−
4	*walK*	Histidine kinase	687783–689081	+
5	*pgsA*	CDP-diacylglycerol-glycerol-3-phosphate-3-phosphatidyltransferase	1627067–1627615	+
6	*bcrA*	ATP-binding protein	1674978–1675691	−
7	*yojI*	ATPase component	1739261–1740082	−
8	*arnA*	Methionyl-tRNA formyltransferase	2166583–2168565	−
9	*pmrF*	Phosphotransferase	2168565–2169545	−
10	*basR*	Response regulator	2491364–2492029	+
11	*lpxC*	N-acetylglucosamine deacetylase	2834499–2835419	−
12	*rosB*	Kef family K (+) transporter	3000038–3001798	+
13	*cdsA*	Phosphatidate cytidylyltransferase	3018284–3019219	+

^a^ Location information of genes in the P3M genome (5′–3′). ^b^ Genes are located in the positive (+) or negative (−) strand of the P3M genome.

**Table 4:** The optimal secondary structures upon hybridization between candidate ncRNAs and possible target mRNAs.

Candidate ncRNA	Possible Target mRNA	Binding Sites on ncRNA (5′–3′)	Stem–Loop Structure ^a^	Binding Sites on Target mRNA (5′–3′)	Post-Transcriptional Translation Process ^b^
ncRNA31	*liaR* mRNA	35 to 42	T	342 to 349	N
	*pgsA* mRNA	19 to 26	T	39 to 46	N
	*yojI* mRNA	58 to 68	T	224 to 234	N
	*basR* mRNA	19 to 22	F	106 to 109	N
ncRNA34	*liaR* mRNA	63 to 68	T	31 to 37	N
	*pgsA* mRNA	62 to 68	T	537 to 543	P
	*yojI* mRNA	44 to 64	T	756 to 776	P
	*basR* mRNA	63 to 67	T	316 to 320	N
ncRNA40	*liaR* mRNA	37 to 41	T	230 to 234	N
	*pgsA* mRNA	33 to 41	T	5 to 13	N
	*yojI* mRNA	31 to 40	T	9 to 18	N
	*basR* mRNA	38 to 45	T	230 to 237	N
ncRNA45	*liaR* mRNA	130 to 142	T	131 to 145	N
	*pgsA* mRNA	103 to 119	T	90 to 105	P
	*yojI* mRNA	95 to 104	T	255 to 264	P
	*basR* mRNA	66 to 73	T	90 to 97	N
ncRNA46	*liaR* mRNA	99 to 108	T	118 to 127	N
	*pgsA* mRNA	2 to 23	F	330 to 352	N
	*yojI* mRNA	91 to 111	T	81 to 99	N
	*basR* mRNA	11 to 22	F	90 to 105	N
ncRNA58	*liaR* mRNA	38 to 43	F	416 to 421	N
	*pgsA* mRNA	17 to 20	T	492 to 495	N
	*yojI* mRNA	17 to 20	T	695 to 698	N
	*basR* mRNA	14 to 15	F	567 to 568	N

^a^ T/F refers to the interaction sites that are/are not located on the typical stem–loop structure of ncRNA. ^b^ P/N refers to the binding of ncRNA to target mRNA that promotes/does not promote the unfolding of the target mRNA secondary structure near the interaction sites.

**Table 5:** Determination of minimal inhibitory concentrations (MICs) of polymyxin B on P3M.

Strains	MIC (mg/L), Interpretation
P3M	>1024, Resistant
∆ncRNA34	1024, Resistant
com-ncRNA34	>1024, Resistant

## Data Availability

The original contributions presented in the study are included in the article/supplementary material, further inquiries can be directed to the corresponding author.

## Supplementary Materials

The following supporting information can be downloaded at: https://www.mdpi.com/article/10.3390/microorganisms12081661/s1, Figures S1–S6: Folding forms of ncRNA31, ncRNA34, ncRNA40, ncRNA45, ncRNA46, and ncRNA58 based on minimum free energy, respectively; Figures S7–S10: The corresponding interaction sites of ncRNAs on the possible *liaR* mRNA, *pgsA* mRNA, *yojI* mRNA, and *basR* mRNA with distinct colors at their respective positions, respectively; Table S1: Primers used in this study.
