# Supplementary figures and images for: Identification and Functional Analysis of ncRNAs Regulating Intrinsic Polymyxin Resistance in Foodborne Proteus vulgaris
Source: Microorganisms. 2024 Aug 13;12(8):1661. doi: 10.3390/microorganisms12081661 (PMC11356903; doi:10.3390/microorganisms12081661)

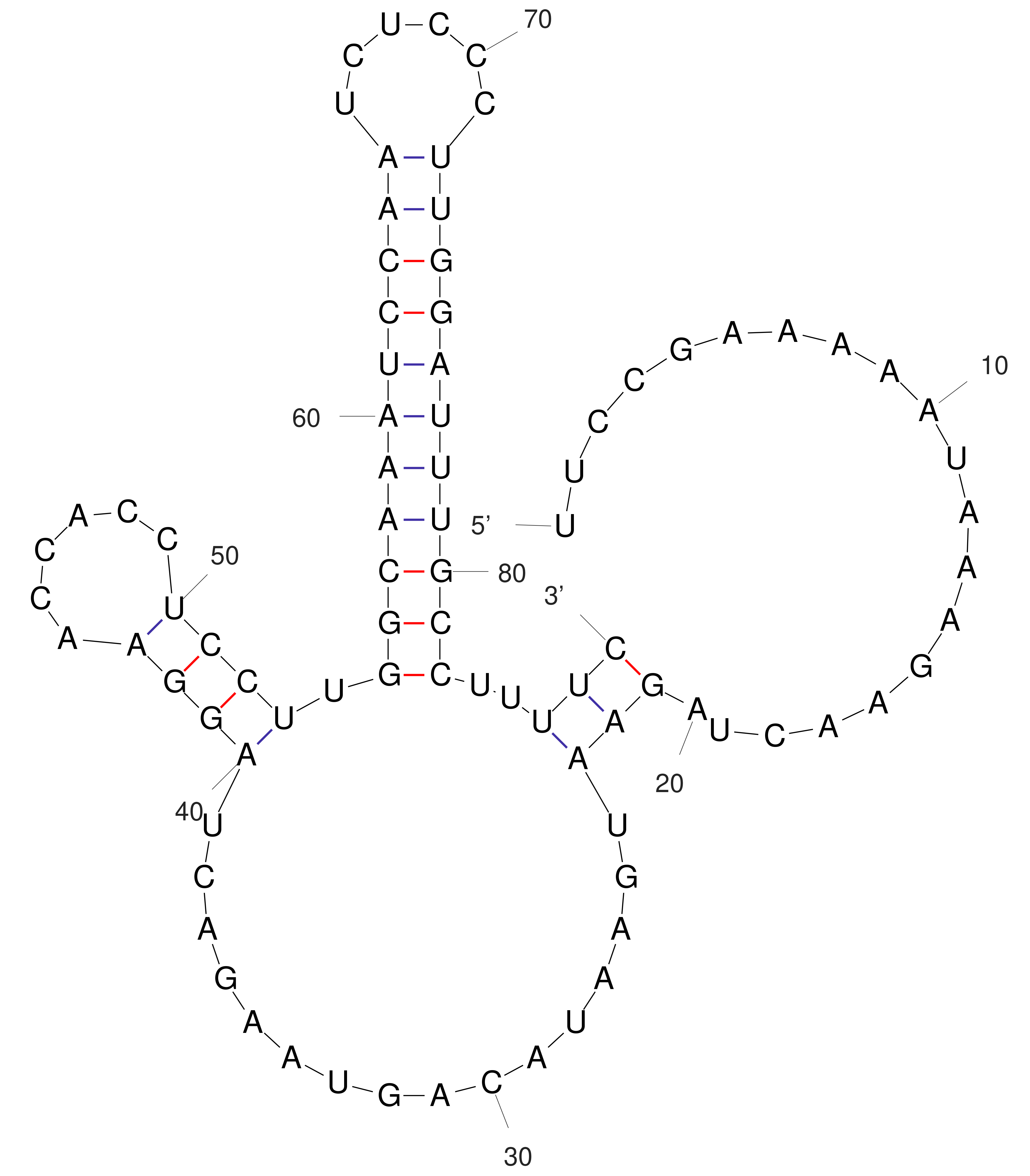

Supplement: Supplementary file 1 [file microorganisms-12-01661-s001.zip › Figure S1.jpg]

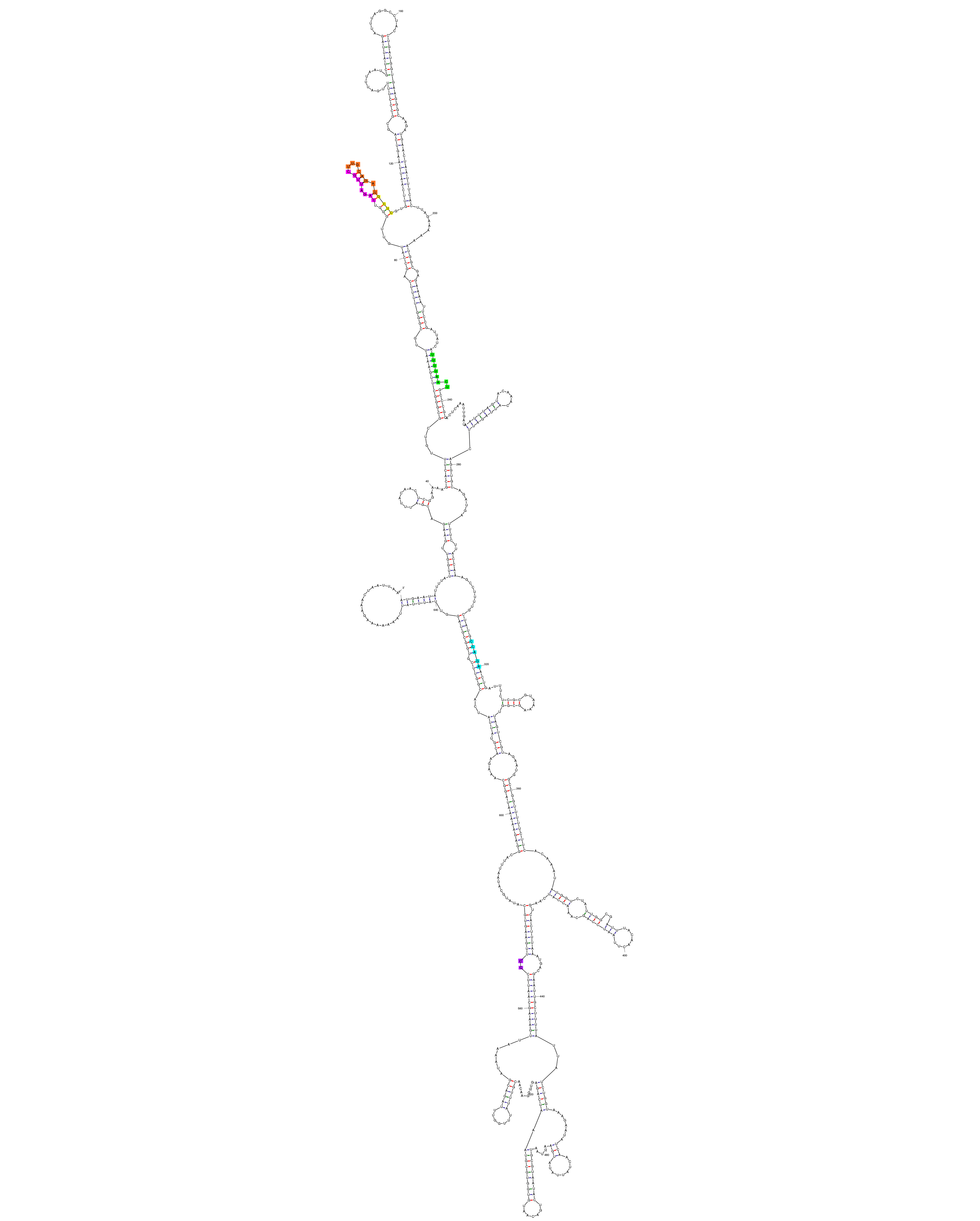

Supplement: Supplementary file 1 [file microorganisms-12-01661-s001.zip › Figure S10.jpg]

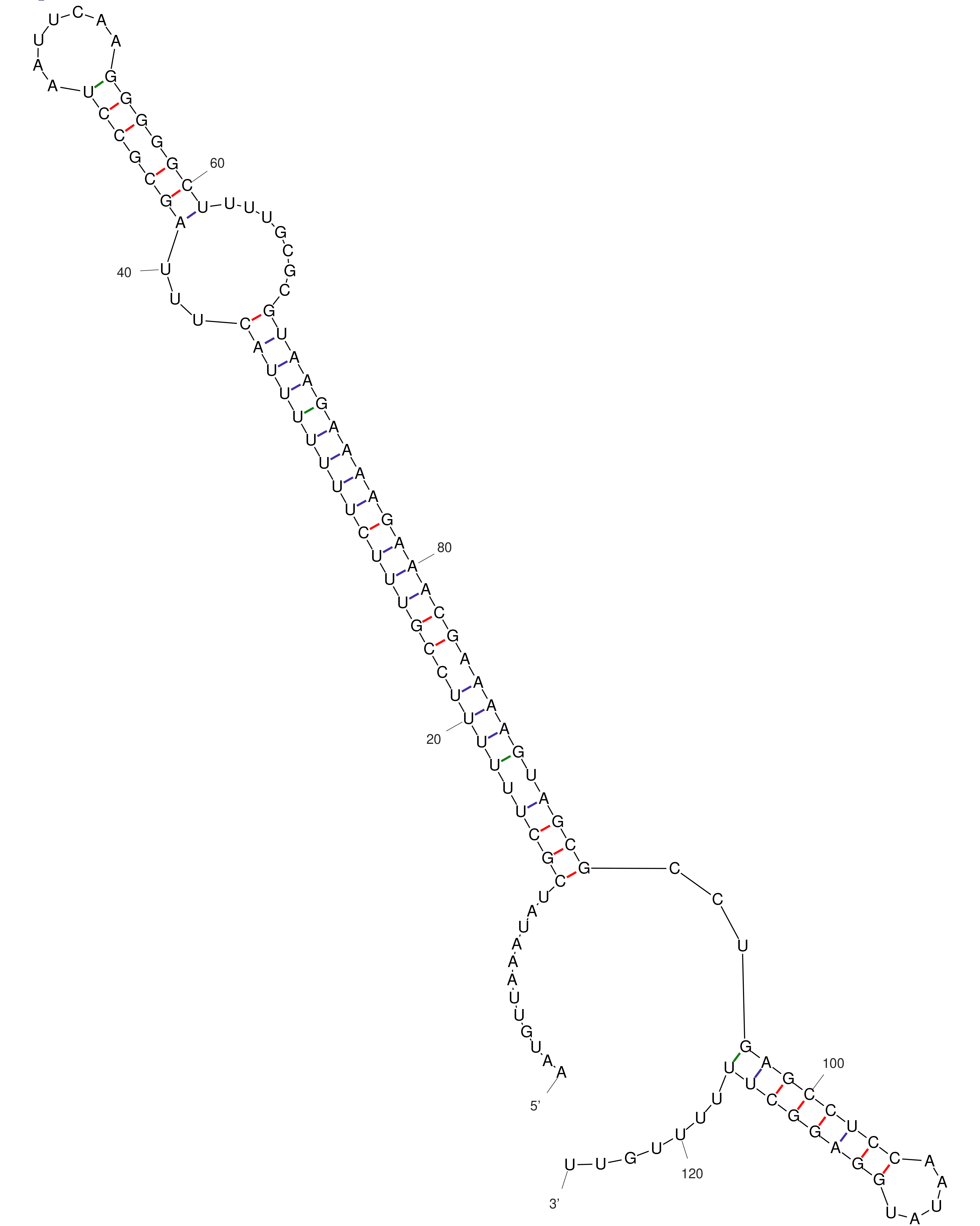

Supplement: Supplementary file 1 [file microorganisms-12-01661-s001.zip › Figure S2.jpg]

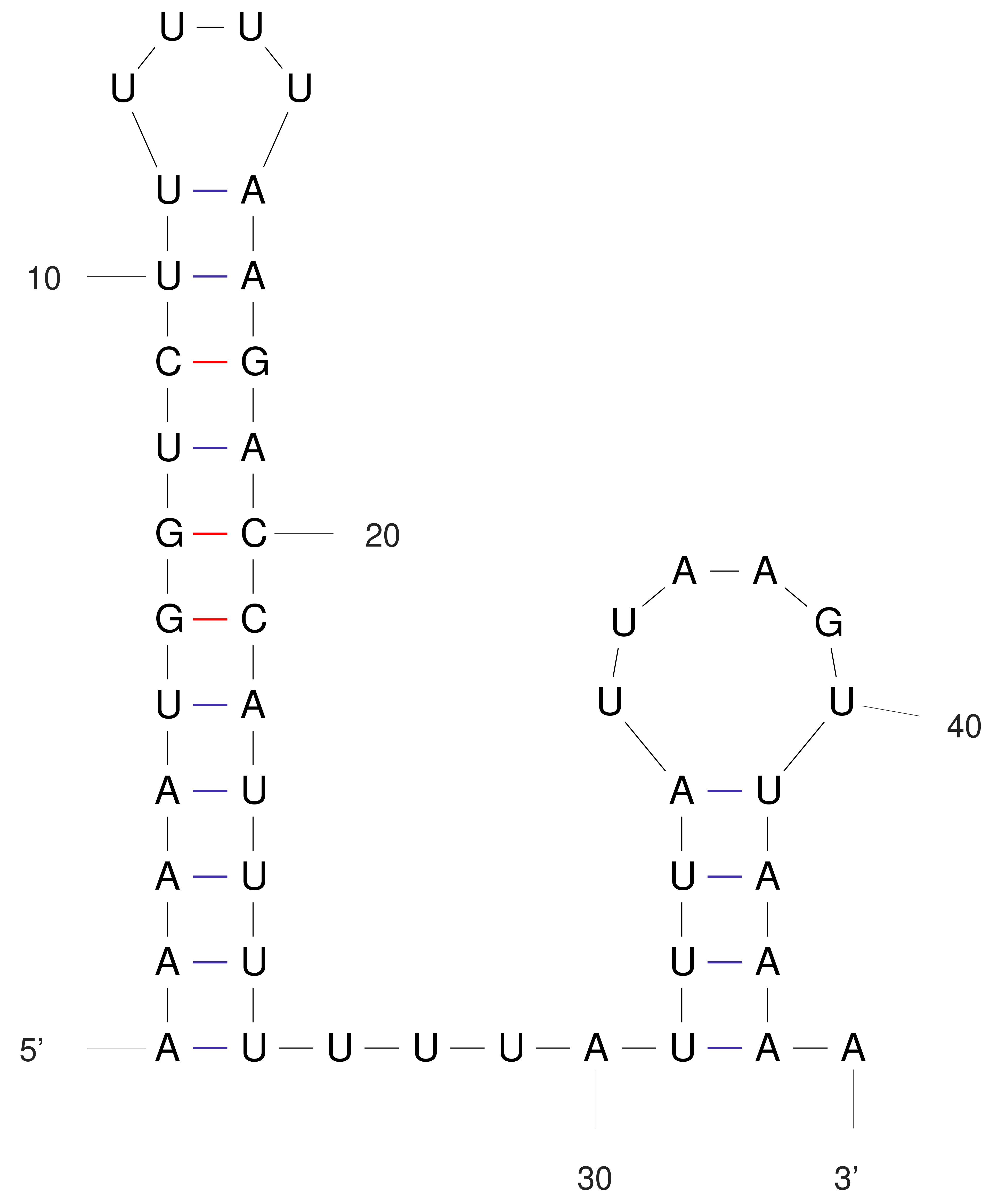

Supplement: Supplementary file 1 [file microorganisms-12-01661-s001.zip › Figure S3.jpg]

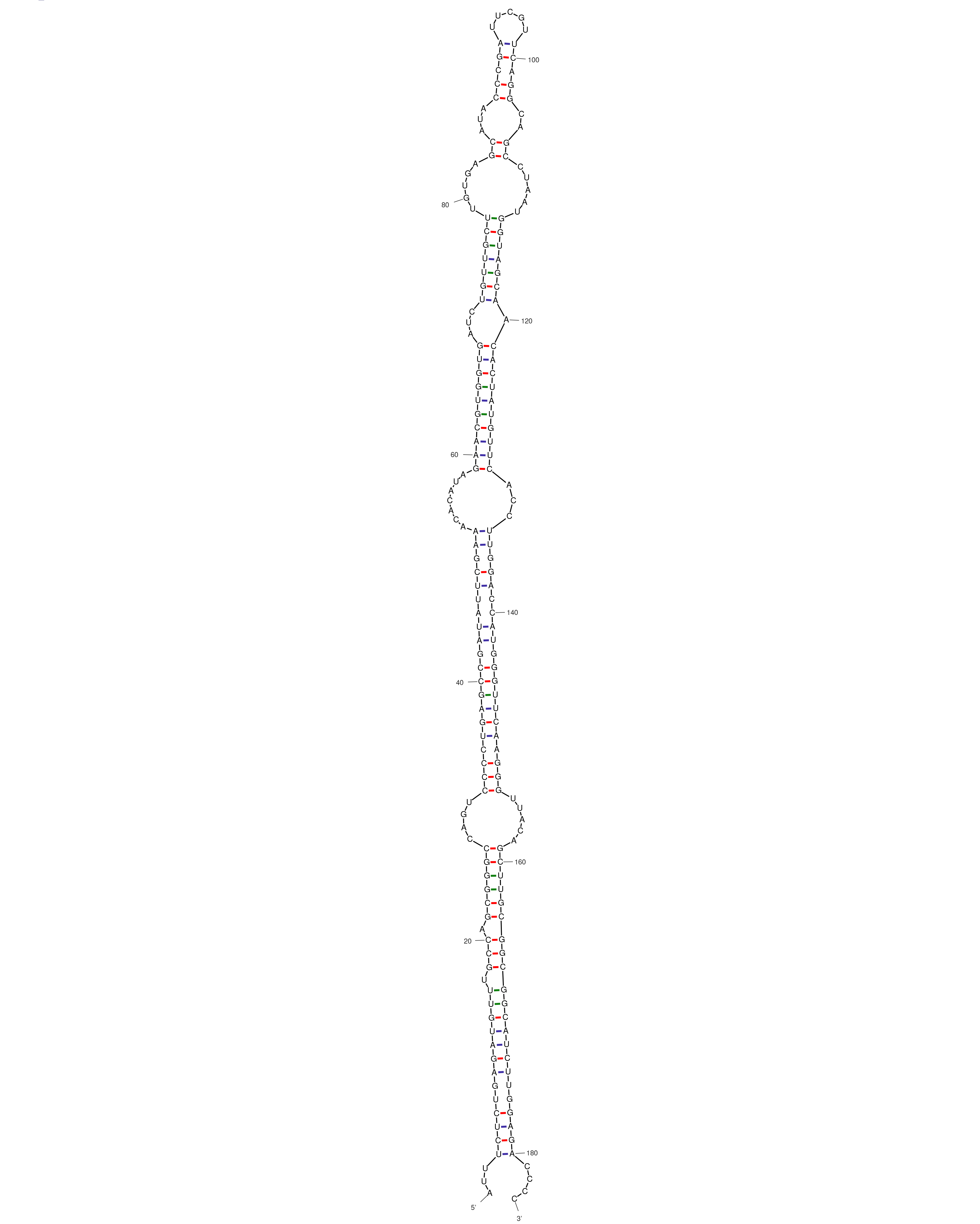

Supplement: Supplementary file 1 [file microorganisms-12-01661-s001.zip › Figure S4.jpg]

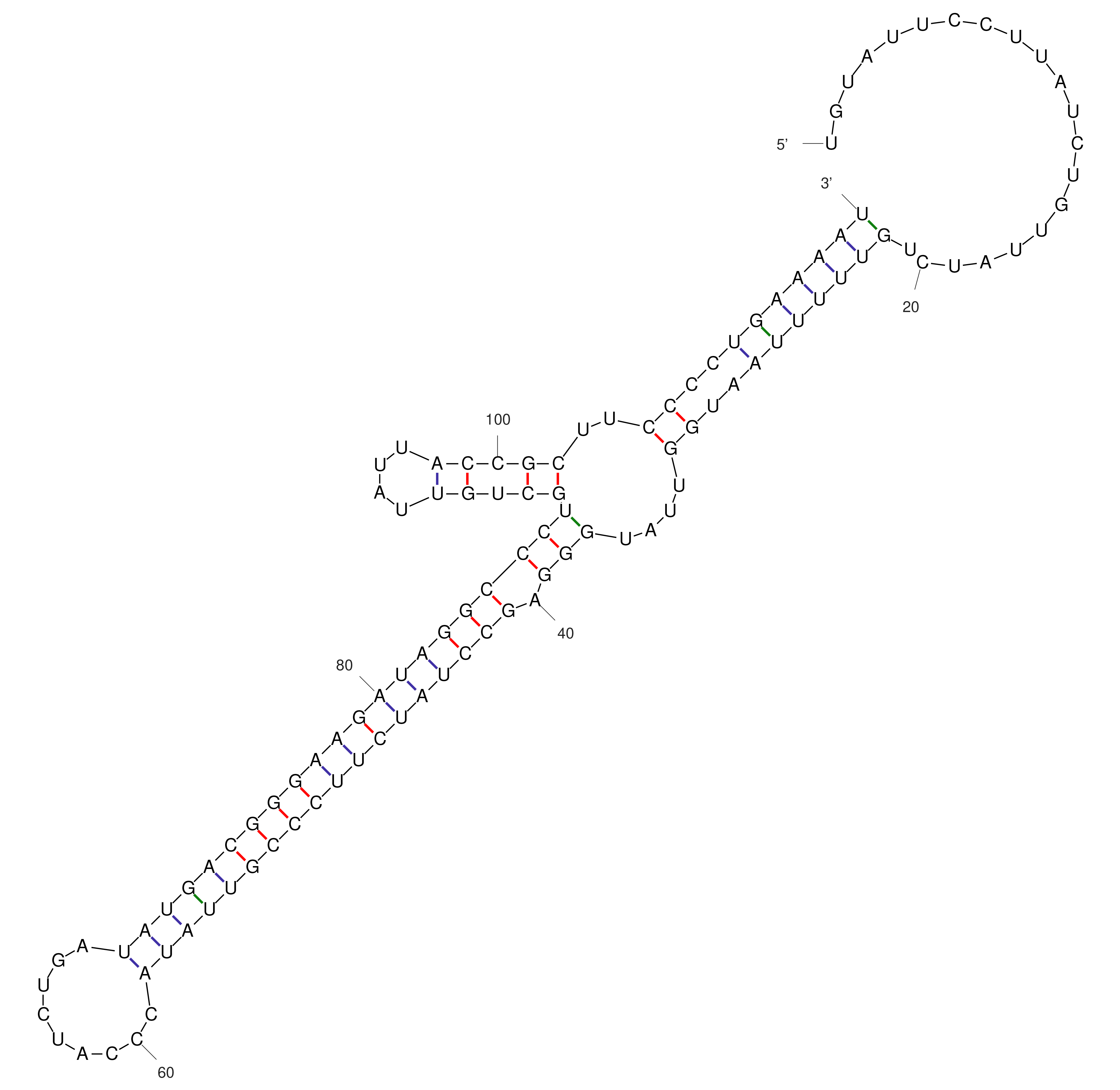

Supplement: Supplementary file 1 [file microorganisms-12-01661-s001.zip › Figure S5.jpg]

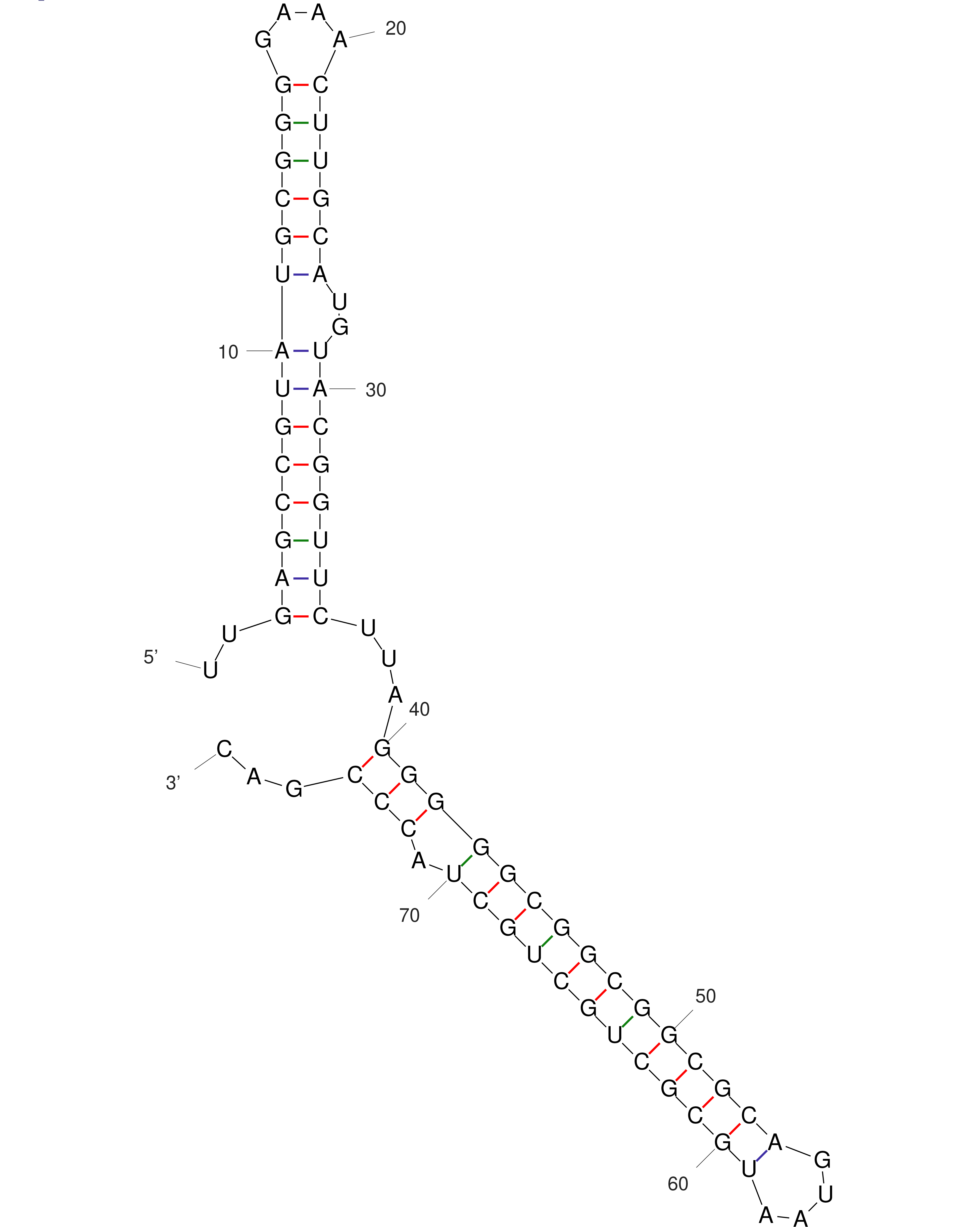

Supplement: Supplementary file 1 [file microorganisms-12-01661-s001.zip › Figure S6.jpg]

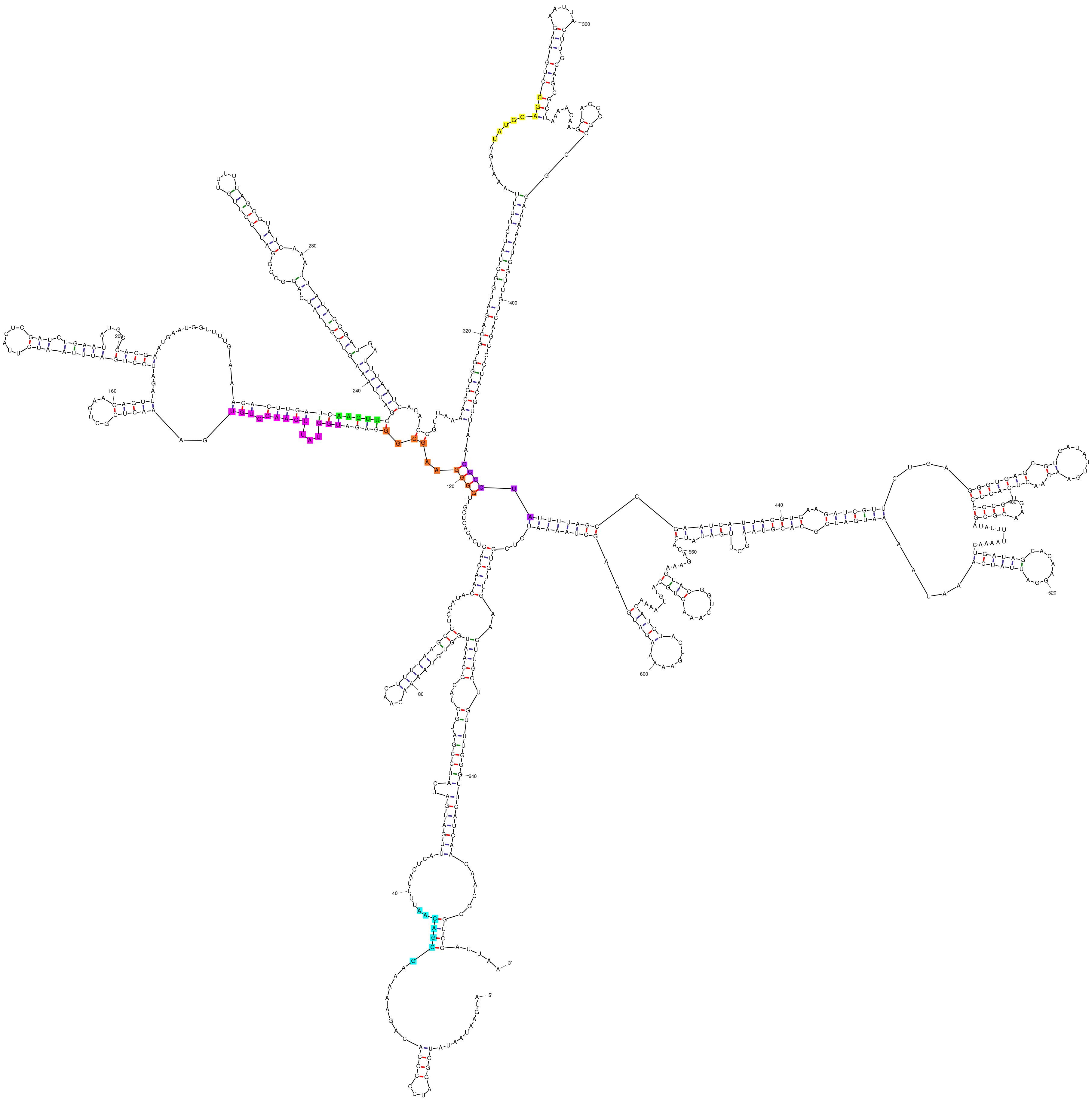

Supplement: Supplementary file 1 [file microorganisms-12-01661-s001.zip › Figure S7.jpg]

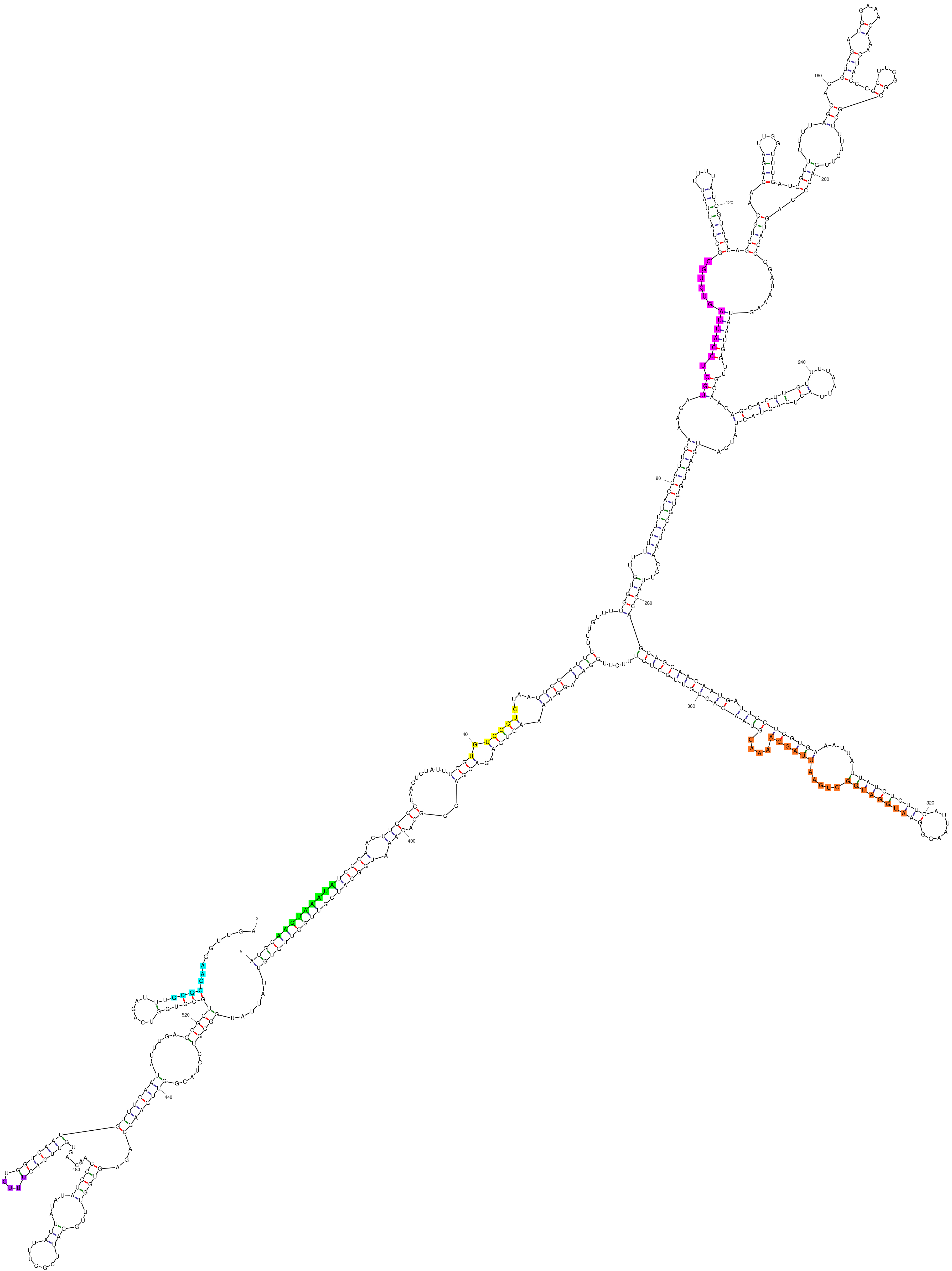

Supplement: Supplementary file 1 [file microorganisms-12-01661-s001.zip › Figure S8.jpg]

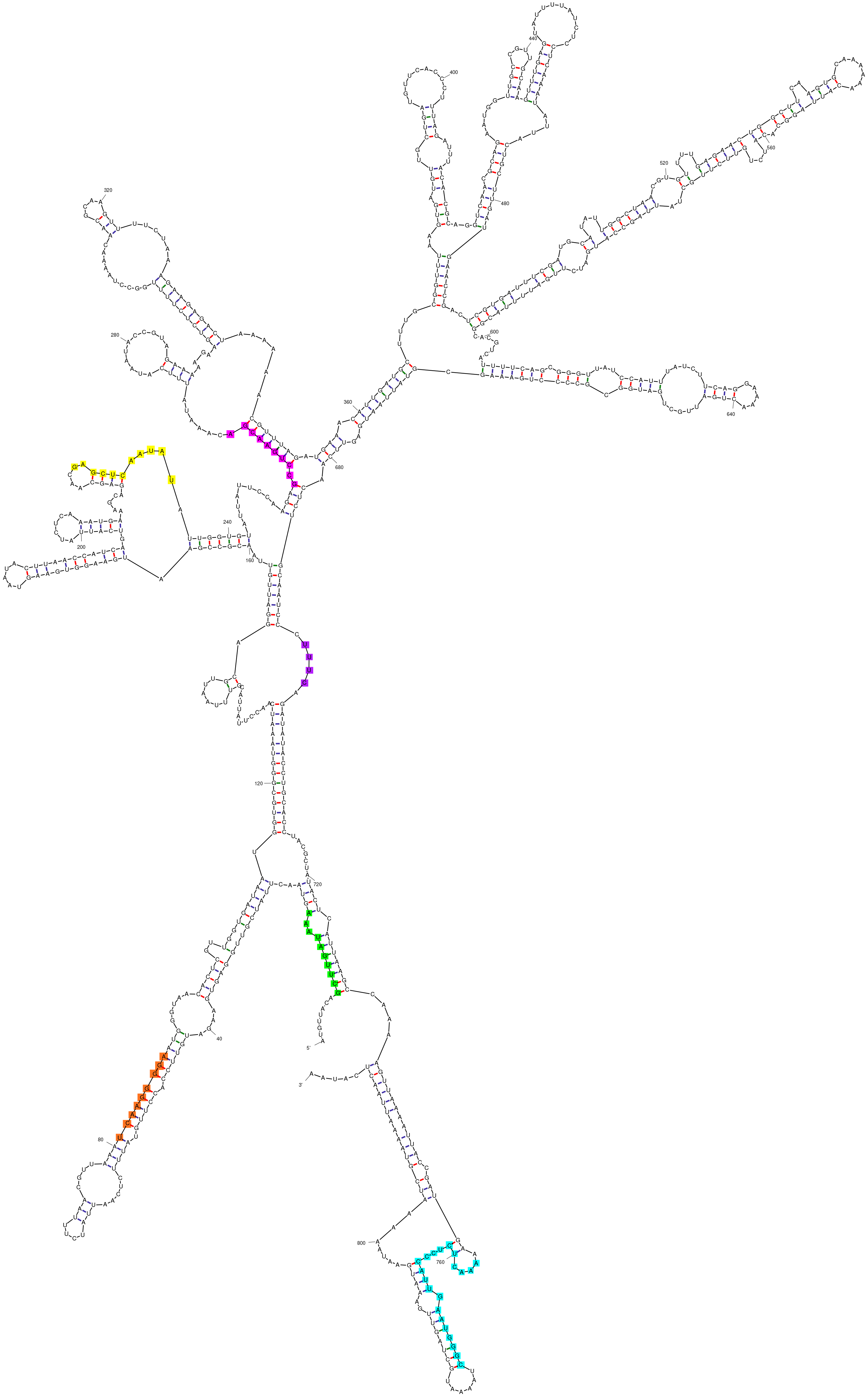

Supplement: Supplementary file 1 [file microorganisms-12-01661-s001.zip › Figure S9.jpg]
